# From physical activity patterns to cognitive status: development and validation of novel digital biomarkers for cognitive assessment in older adults

**DOI:** 10.1186/s12966-025-01706-x

**Published:** 2025-01-20

**Authors:** Ling-Jie Fan, Feng-Yi Wang, Jun-Han Zhao, Jun-Jie Zhang, Yang-An Li, Jia Tang, Tao Lin, Quan Wei

**Affiliations:** 1 College of Computer Science, Sichuan University, Chengdu, China; 2 Department of Rehabilitation Medicine, West China Hospital of Sichuan University, Chengdu, China; 3 Department of Biomedical Informatics, Harvard Medical School, Boston, MA USA; 4 Department of Biostatistics, Harvard T.H. Chan School of Public Health, Boston, MA USA

**Keywords:** Physical activity, Cognitive function, Accelerometer, Explainable machine learning

## Abstract

**Background:**

This study aims to investigate the associations between signal-level physical activity (PA) features derived from wrist accelerometry data and cognitive status in older adults, and to evaluate their potential predictive value when combined with demographics.

**Methods:**

We analyzed PA data from 3,363 older adults (NHATS: *n* = 747; NHANES: *n* = 2,616), with each participant contributing a complete 3-day continuous activity sequence. We extracted the most relevant PA features associated with cognitive function using feature engineering and recursive feature elimination. Demographic characteristics were also incorporated, and multimodal data fusion was achieved through canonical correlation analysis. We then developed explainable machine learning models, primarily random forest, optimized with hyperparameters, to predict individual cognitive function status.

**Results:**

Using recursive feature elimination, we identified the top 20 PA features from each dataset and combined them with demographic features for modeling. The models achieved AUCs of 0.84 and 0.80 for NHATS and NHANES. Change quantiles and FFT coefficients emerged as the consistently top-ranked PA features across datasets, ranking 1st and 2nd respectively in their predictive importance for cognitive function. Models based on the top 10 PA features common to both datasets, along with demographic features, achieved AUCs above 0.8.

**Conclusions:**

This study identifies novel time-frequency domain features of physical activity that show robust associations with cognitive status across two independent cohorts. These features, particularly those capturing activity variability and rhythmicity, provide complementary information beyond traditional cumulative PA measures. Based on these findings, we developed a proof-of-concept application that demonstrates the feasibility of translating these PA analytics into practical monitoring tools in real-world settings.

**Supplementary Information:**

The online version contains supplementary material available at 10.1186/s12966-025-01706-x.

## Background

As the global population ages, the challenge of cognitive impairment is becoming increasingly severe. In the United States, cognitive impairment has emerged as a major issue affecting the health and quality of life of older adults [1]. It not only significantly impacts daily life and independent functioning but also places a substantial economic burden on families and society [2]. Real-time monitoring of cognitive function in the aging population and early screening and intervention are crucial for improving quality of life, reducing societal burden [3] and against the dementia tsunami [4].

However, traditional approaches for assessing cognitive status such as neuropsychological tests, neuroimaging, and serum or cerebrospinal fluid markers, have inherent limitations. These approaches are often time-consuming, expensive, and require specialized medical support, limiting their accessibility and practicality [5]. Even brief cognitive assessments require healthcare professional involvement and cannot be performed frequently due to practice effects and the need for standardized testing conditions.

PA has been linked to cognitive function through multiple epidemiological and intervention studies. A large-scale cohort study (*n* = 2,700) found that older adults engaging in ≥ 150 min/week of moderate-to-vigorous physical activity (MVPA) showed 36% lower risk of cognitive decline over a 5-year follow-up period [6]. Similarly, data from the English Longitudinal Study of Ageing (*n* = 3,400) revealed that participants maintaining ≥ 120 min/week of MVPA demonstrated better executive function and memory performance compared to those who were less active [7]. A meta-analysis of 36 studies further suggested that even light-intensity activities, accumulated to ≥ 300 min/week, were associated with a 23% reduction in cognitive decline risk [8].

However, these findings primarily rely on subjective PA questionnaires or activity logs, which show limited reliability (*r* = 0.3–0.5) when compared to objective measures and are susceptible to recall bias, particularly in older populations [9]. For instance, the correlation between self-reported and accelerometer-measured MVPA time was only 0.4 in adults over 65 years [10].

With advances in wearable technology, accelerometer-based PA monitoring has emerged as an objective approach for characterizing physical activity patterns. Wrist-worn accelerometers can capture continuous activity data at sampling rates of 30–100 Hz, providing detailed information about activity intensity, duration, and patterns throughout the day [11]. These technological capabilities enable us to build a more sophisticated bridge connecting accelerometer signals to behavioral patterns, and ultimately to cognitive function. Through advanced signal processing and pattern recognition techniques, we can potentially identify specific PA signatures that may reflect underlying cognitive status, moving beyond simple activity duration measures to more nuanced characterizations of daily functioning.

Recent studies using accelerometer data have primarily focused on cumulative indicators (e.g., daily average PA and sedentary time) in relation to cognitive status [12]. While these studies provide valuable insights, they may not fully capture the rich temporal and frequency characteristics inherent in continuous PA data. PA patterns contain complex information about variability, rhythmicity, and complexity that could potentially reflect different aspects of daily functioning [13]. Furthermore, these activity patterns are influenced by multiple factors, including demographic characteristics such as race [14] and clinical conditions [15], suggesting the need for a more comprehensive analytical approach.

Analyzing these complex activity patterns presents several methodological challenges. First, the high-dimensional nature of accelerometer time series data requires sophisticated signal processing and feature extraction techniques to identify meaningful patterns. Second, the integration of multiple data types (PA patterns, demographics, and health information) necessitates advanced statistical approaches to handle their complex interactions [16]. Additionally, while machine learning methods can effectively process such complex data, ensuring model interpretability remains crucial for clinical applications [17].

This study aimed to: (1) identify novel time-frequency domain features from accelerometer data that show consistent associations with cognitive status across two independent cohorts of older adults; (2) examine how these PA features, when combined with demographic and health information, relate to cognitive status; and (3) develop interpretable machine learning models to evaluate the potential utility of these features. We extracted comprehensive PA features from accelerometer data, including temporal patterns, frequency characteristics, and nonlinear properties, beyond traditional cumulative measures. These features were then analyzed alongside demographic and health information using interpretable machine learning approaches to understand their relationships with cognitive status. Based on these analyses, we developed a proof-of-concept application to demonstrate how these PA features could be calculated and monitored in real-world settings using wearable devices.

## Methods

**Fig. 1:**
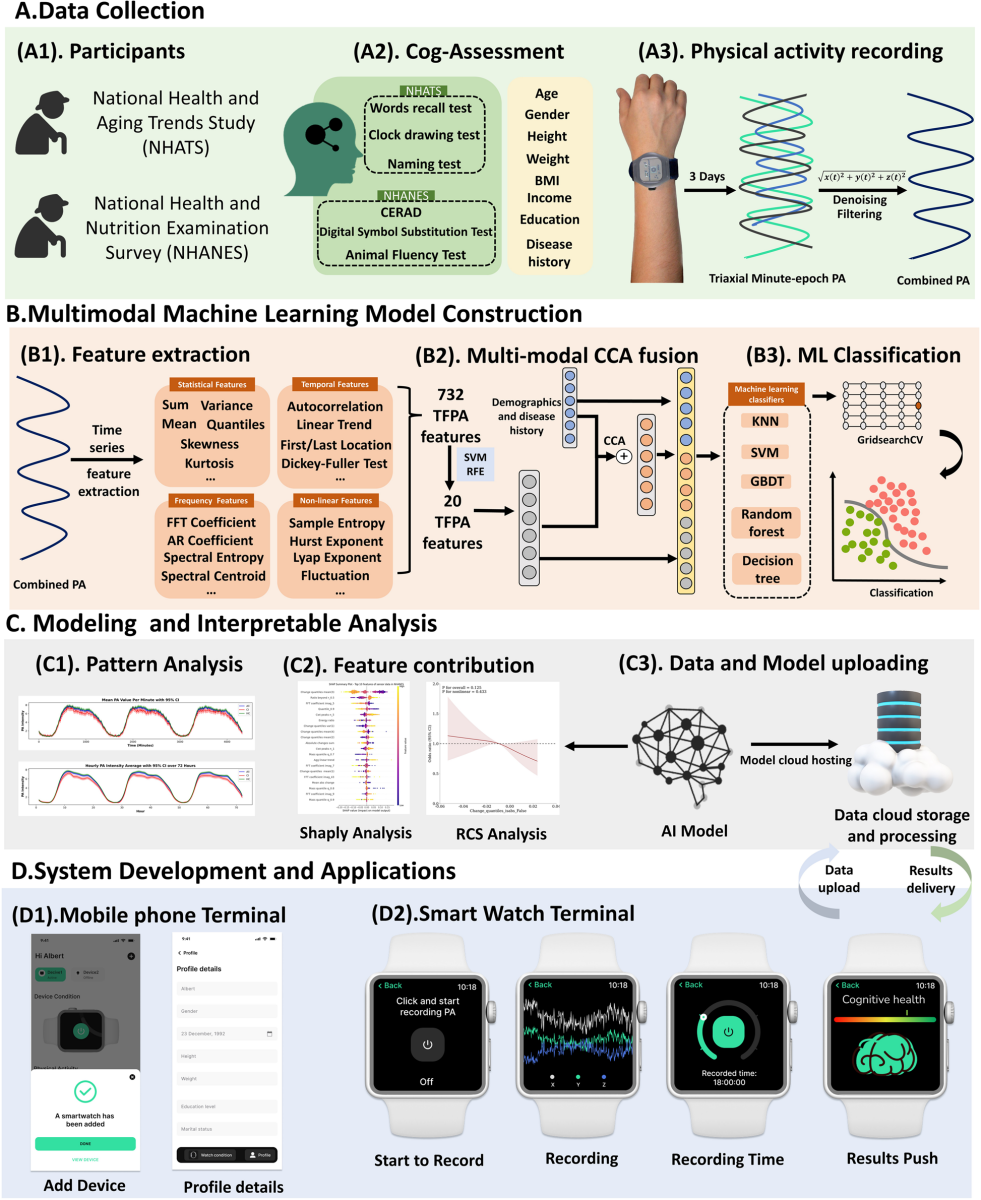
Overview of Experimental design. Figure 1. **A** Data Collection. (A1) Dataset selection: MHATS and NHANES. (A2) Collection of cognitive performance measures and demographic/medical history information (A3) Acquisition and preprocessing of PA data. **B** Construction of Multimodal Machine Learning Models. (B1) Extraction of 777 PA features and dimensionality reduction to 20 based on RFE. (B2) Fusion of PA features and demographic features based on CCA. (B3) Modeling of fused features using 5 machine learning algorithms and hyperparameter optimization based on GridSearchCV. **C** Model Analysis. (C1) Visual analysis of PA data. (C2) Feature contribution analysis based on SHAP and correlation analysis based on RCS (C3) Deployment of models to the cloud. **D** Development of Mobile System (D1) Mobile phone interface: used for filling in basic information and connecting devices. (D2) Smartwatch interface: used for monitoring control and result push notifications

This study analyzed accelerometer data from two large-scale cohort studies of older adults: the National Health and Aging Trends Study (NHATS) and the National Health and Nutrition Examination Survey (NHANES). Our analytical framework consisted of three main components (Fig. 1): (1) data preprocessing and feature extraction from accelerometer signals, (2) development and validation of interpretable machine learning models incorporating both movement features and demographic characteristics, and (3) comprehensive model evaluation using multiple statistical approaches. Raw accelerometer data were processed to extract time-frequency domain features that capture various aspects of movement patterns. We initially derived 777 features describing temporal patterns, frequency characteristics, and nonlinear properties of the movement signals. These features were then systematically evaluated using recursive feature elimination (RFE) to identify the most robust features associated with cognitive status. Demographic and health information were integrated with the selected movement features through Canonical Correlation Analysis (CCA). Multiple machine learning models were developed and compared, with particular attention to model interpretability through Shapley Additive Explanations (SHAP) and feature-outcome relationships through Restricted Cubic Splines (RCS) analysis. All models were independently validated in both cohorts to assess generalizability.

### Participants

This study utilized data from two national studies in the United States: the National Health and Aging Trends Study (NHATS) and the National Health and Nutrition Examination Survey (NHANES). NHATS, funded by the National Institute on Aging (NIA), is a longitudinal cohort study that comprehensively assesses changes in health status, physical performance, and social environment among older adults through annual face-to-face interviews and examinations [18]. NHANES, conducted by the Centers for Disease Control and Prevention, is a continuous national health survey that has monitored the health and nutritional status of the U.S. population since the 1960s through questionnaires, physical examinations, and laboratory tests [19].

From the NHATS Round 11 (2021) sample of 3,817 community-dwelling older adults, 747 participants had complete accelerometer and cognitive data for analysis. Participants were excluded if they had incomplete cognitive assessments (*n* = 429) or insufficient accelerometer wear time (*n* = 2,641). From the NHANES 2011–2014 cycles (*n* = 9,757), 2,616 participants met the inclusion criteria after excluding those with incomplete cognitive data (*n* = 16,460) or insufficient accelerometer data (*n* = 857). The flow of participant selection is detailed in Supplementary Fig. 1.

To ensure data quality and analytical rigor, we established strict inclusion criteria. Participants were required to have complete 3-day accelerometer recordings (72 consecutive hours from 00:00 of day 1 to 24:00 of day 3), with each day containing ≥ 10 h of valid wear time. Additionally, participants needed complete data for all demographic variables and cognitive assessments. After applying these criteria, our final analytical sample included 3,363 participants (NHATS: *n* = 747; NHANES: *n* = 2,616), with each participant contributing exactly 4,320 min-epoch data points.

### Cognitive function

In NHATS, cognitive status was evaluated using a comprehensive approach that included three components: (1) self- or proxy-reported physician diagnosis of dementia or Alzheimer’s disease; (2) the AD8 dementia screening interview [20] administered to proxies, with a score of ≥ 2 indicating possible cognitive impairment; and (3) a cognitive test battery assessing memory (immediate and delayed word recall), orientation (date, month, year, and day of the week), and executive function (clock drawing test). Based on established criteria [21], participants were classified as having possible cognitive impairment if they met any of the following: confirmed physician diagnosis, AD8 score ≥ 2, or scores falling below 1.5 standard deviations of the age-adjusted mean in at least two cognitive domains for self-respondents.

NHANES (2011–2014) administered three validated cognitive assessments to adults aged 60 and older: the Consortium to Establish a Registry for Alzheimer’s Disease (CERAD) Word Learning Test for immediate and delayed recall [22], the Animal Fluency Test (AFT) for semantic fluency [23], and the Digit Symbol Substitution Test (DSST) for processing speed and executive function [24]. Following established protocols for identifying lower cognitive performance in population-based studies [25], we defined lower cognitive performance as scoring at or below age- and education-adjusted 25th percentile. This approach identifies individuals who may benefit from further cognitive evaluation, though it does not constitute a clinical diagnosis.

### PA data from wrist accelerometer

Both NHATS and NHANES datasets utilized wrist-worn accelerometers to continuously monitor participants’ PA levels for multiple days, 24 h a day. In NHATS, participants wore an Actigraph CentrePoint Insight Watch for 7 days following their in-home interview, with a sampling frequency of 64 Hz. Researchers used the ARCTOOLS software package to process the data, summarizing it into 1-minute epochs and defining valid days as those with more than 1296 min of wear time [26]. Similarly, NHANES participants wore an ActiGraph GT3X + accelerometer for 7 consecutive days, collecting triaxial acceleration data at 80 Hz and ambient light data once per second. The raw data were aggregated into minute-level data for each participant during post-processing.

Despite differences in accelerometer models, sampling frequencies, and target populations, both studies employed consistent PA monitoring protocols, requiring participants to wear the devices continuously for multiple days. This approach allowed for the collection of unified minute-level PA intensity sequences, facilitating the application of machine learning algorithms to both datasets.

Our analysis included 3,363 participants (NHATS: *n* = 747; NHANES: *n* = 2,616), treating each participant’s 3-day continuous sequence (4,320 min-epochs) as an indivisible unit. For data preprocessing, we performed dimension merging on the triaxial PA data from NHANES using the sum of squares operation and applied a Butterworth filter to reduce abnormal noise. Importantly, we maintained the complete temporal sequence for each participant without any segmentation, ensuring the preservation of continuous activity patterns. This participant-level approach, combined with our strict cross-validation strategy that keeps all data from the same participant exclusively in either training or testing sets, prevents data leakage and enhances the robustness of our validation process.

### PA pattern analysis from accelerometer data

Traditional analyses of accelerometer data often focus on cumulative measures such as total activity counts or total time spent in different intensity levels over a day. While these cumulative metrics are valuable, they alone may not fully capture the dynamic patterns of physical activity. For instance, two individuals might have identical total daily activity counts but achieve them through fundamentally different activity patterns [26].

To characterize PA patterns comprehensively at the participant level, we analyzed each participant’s complete 3-day sequence using four complementary approaches: statistical, temporal, frequency, and nonlinear. Using signal processing methods from tsfresh, we extracted 777 features from each participant’s complete minute-level PA sequence, ensuring that all temporal dependencies within individual sequences were preserved. These features capture different properties of the entire 3-day PA sequence and reflect multiple dimensions of individual PA behavior. Each feature category describes distinct properties of the complete PA sequence from different perspectives, representing specific activity patterns that may be closely related to cognitive function. The specific features in each category are detailed below:

Statistical features describe the basic statistical properties of PA data and reflect the distribution characteristics of PA intensity.

#### Sum and mean

Represent the total amount and average level of PA, indicating an individual’s overall activity level.

#### Skewness and kurtosis

Describe the asymmetry and tail characteristics of the PA intensity distribution. High skewness indicates a distribution with a long right tail, suggesting that the individual tends to engage in more high-intensity PA; high kurtosis indicates a heavy-tailed distribution, suggesting that extreme-intensity PA is more frequent.

#### Count below mean

Represents the number of PA instances below the average intensity, reflecting the frequency of an individual engaging in low-intensity activities.

Temporal features capture the dynamic change characteristics of PA time series and reflect the organizational patterns of PA in the time dimension.

#### Linear Trend

Represents the linear trend of the PA time series, reflecting the long-term change trend of an individual’s PA level.

#### Change quantiles

Describes the quantile features of the change magnitude in the PA time series, reflecting the intensity of an individual’s PA fluctuations and further representing the diversity of activities.

Frequency domain features describe the characteristics of PA time series in the frequency dimension, reflecting the periodicity and rhythmicity of PA.

#### FFT Coefficient

(Fast Fourier Transform Coefficient): Represents the energy distribution of the PA time series across different frequency components, reflecting the periodic characteristics of an individual’s PA.

#### AR Coefficient

(Autoregressive Coefficient): Describes the autoregressive properties of the PA time series, reflecting the time-dependence of an individual’s PA behavior.

Nonlinear features capture the complex dynamic properties of PA time series, reflecting the nonlinearity and unpredictability of PA behavior.

#### Sample entropy

Measures the complexity of the PA time series. Higher sample entropy suggests that PA behavior is more random and unpredictable, which may be related to cognitive flexibility.

In summary, the typical PA features from the four categories describe the multidimensional attributes of individual activity behavior from different perspectives, represent specific activity patterns, and may be associated with cognitive function through various mechanisms.

### Demographics and medical information

Cognitive function and activity behavior are influenced by various factors, including genetics, environment, and lifestyle [27]. In this study, we included demographic and disease history variables as covariates due to their accessibility and universality, rather than biochemical or imaging indicators. We selected a consistent set of covariates for both the NHATS and NHANES datasets to ensure comparability.

Demographic variables included age, gender, race, height, weight, body mass index (BMI), education level, marital status, and wage income. Disease history incorporated prevalent chronic conditions such as heart attack, heart disease, stroke, hypertension, diabetes, arthritis and lung disease. In addition to this, due to the potential relationship between mood and cognitive functioning [28], we included mood status calculated based on PHQ9 (NHANES) versus PHQ4 (NHATS), all of which have been identified as risk factors for cognitive decline and may impair an individual’s ability to engage in activities.

### Statistical analysis

Our analytical approach aimed to identify PA patterns associated with cognitive status and evaluate their potential predictive value when combined with demographic characteristics. The analysis proceeded in four main steps:

Firstly, seven hundred and seventy-seven features from the PA data were computed based on the tsfresh library. Missing values in demographic and disease history variables were imputed using the Missforest algorithm [29] to improve data quality and model performance. PA data were visualized using time series plots to illustrate differences in PA patterns among individuals with varying cognitive levels. The Random Forest-based RFE method was used to select the top 20 important features from the 777 PA features.

Secondly, to fully utilize the inter-correlated information between PA features and demographic and disease history features, we introduced a feature fusion method based on CCA [30]. CCA learns a common subspace by maximizing the correlation between features from different modalities, achieving fusion between modalities. Given two feature matrices $\:{X}_{1}$ and $\:{X}_{2}$ from different modalities, the objective of CCA is to find two linear transformations $\:{w}_{1}$ and $\:{w}_{1}$ that maximize the correlation between the transformed features:

1 $$\:{max}_{{w}_{1},{w}_{2}}=\frac{{w}_{1}^{T}{X}_{1}{X}_{2}^{T}{w}_{2}}{\sqrt{{w}_{1}^{T}{X}_{1}{X}_{1}^{T}{w}_{1}}\sqrt{{w}_{2}^{T}{X}_{2}{X}_{2}^{T}{w}_{2}}}$$

By solving the above optimization problem, we can obtain a pair of Canonical Projection Vectors (CPV), denoted as:

2 $$\:{v}_{1}={X}_{1}^{T{w}_{1}},\:{v}_{2}={X}_{2}^{T{w}_{2}}$$

We performed CCA fusion on the PA features and demographic and disease history features separately, obtained a set of CPV, and then fused them with the original features to construct a more comprehensive and robust feature set. The number of canonical projection vectors was determined by the minimum dimensionality principle, which means we selected the minimum dimension between the two modalities being fused to ensure optimal information preservation while avoiding overfitting.

Thirdly, a comprehensive two-stage modeling approach was adopted to ensure robust and generalizable results (Supplementary Fig. 2). In the first stage, we randomly split participants (with their complete 3-day activity sequences) into training (80%) and testing (20%) sets, stratified by cognitive status. This participant-level split ensures that all data from the same individual remained in either the training or testing set, preventing any potential data leakage. For each dataset (NHATS and NHANES), separate models were developed using five machine learning algorithms: K-Nearest Neighbors (KNN), Random Forest, Support Vector Machine (SVM), Decision Tree, and Gradient Boosting Decision Tree (GBDT). The models were trained using the top 20 PA features identified through RFE. Hyperparameter optimization was performed using GridSearchCV with 5-fold cross-validation within the training set (parameters of the machine learning algorithm on GridsearchCV are shown in Supplementary Table 1). In the second stage, to develop more generalizable models, we identified the top 10 PA features that showed consistent importance across both datasets based on their SHAP values and re-trained the models using only these common features. This approach ensures that our findings are robust across different populations and measurement conditions. Model performance was evaluated on the held-out test set using multiple metrics including accuracy, AUC-ROC, sensitivity, and specificity. The SHAP method was used to interpret the ML models, providing an intuitive and theoretically supported feature importance measure [31]. This interpretability analysis is crucial for clinical applications and practical decision-making, enhancing the credibility and transparency of the results. SHAP, as a core technology for mining PA digital biomarkers, helps identify the most predictive and interpretable PA features for developing cognitive health monitoring and intervention tools.

Lastly, Univariate analysis using RCS was conducted on the top 10 common PA features to investigate their linear or nonlinear relationships with cognitive function. Pearson correlation coefficients were calculated between PA features, demographic features, and disease history features, and correlation heatmaps were plotted to assess variable correlations. All analyses were conducted using Python 3.10. Statistical significance was set at *p* < 0.05, and 95% confidence intervals were reported where appropriate.

## Results

### Participants characteristics

The baseline characteristics of participants in the NHATS and NHANES datasets are summarized in Table 1.. In the NHATS dataset, the majority of participants in both the healthy control (HC) and cognitive impairment (CI) groups were aged 70–84 years. The gender distribution in the HC group was 46.5% male, while in the CI group, 40.2% were male. Non-Hispanic whites constituted a larger proportion of the HC group (79.8%) compared to the CI group (58.8%). The HC group had a higher average height (1.67 m) and weight (79.66 kg) than the CI group (1.53 m and 73.42 kg, respectively). The prevalence of diabetes and hypertension was lower in the HC group (22% and 57%, respectively) than in the CI group (29% and 67.4%, respectively).

**Table 1:** Demographic characteristics

**Characteristics**	**Attributes**	**NHTAS**		**NHANES**	
		**HC**	**CI**	**HC**	**CI**
**Age**	**60–69**	0	0	855	556
	**70–74**	186	14	244	256
	**75–79**	218	30	121	161
	**80–84**	131	28	140	283
	**85–89**	65	20		
	**≥90**	40	15		
**Gender**	**Male**	298	43	597	673
	**Female**	342	64	763	583
**Race**	**White, non-Hispanic **	531	70	797	475
	**Black, non-Hispanic **	43	16	260	797
	**Others**	66	21	303	563
**Height**		1.67	1.53	1.66	1.65
**Weight**		79.66	73.42	81.45	78.21
**BMI**		28.05	27.1	29.49	28.74
**Diabetes**	**Yes**	141	31	274	341
	**No**	499	76	1086	915
**High blood pressure**	**Yes**	365	72	807	835
	**No**	274	35	553	419
**Stroke**	**Yes**	11	8	61	124
	**No**	628	99	1298	1130
**Heart attack**	**Yes**	11	2	103	125
	**No**	628	104	1257	1131
**Education level**	**High school and below**	211	59	457	813
	**Above high school**	417	44	903	441

In the NHANES dataset, the 60–69 years age group represented a larger proportion of the HC group (47.7%) compared to the CI group (32.9%). The gender distribution was more balanced in the HC group (43.9% male, 56.1% female) than in the CI group (53.6% male, 46.4% female). Non-Hispanic whites accounted for a higher percentage of the HC group (40.9%) than the CI group (27.4%). The average height and weight were similar between the HC group (1.66 m and 81.45 kg, respectively) and the CI group (1.65 m and 78.21 kg, respectively). The prevalence of diabetes was comparable between the HC group (25.2%) and the CI group (27.1%), while the prevalence of hypertension was lower in the HC group (59.4%) than in the CI group (69.2%).

### Visualization of PA Intensity during 3days

To investigate the relationship between PA and cognitive function, we conducted a visual analysis of PA intensity on minute and hourly scales in the NHATS and NHANES datasets (Supplementary Fig. 3). In the NHATS dataset, the HC group consistently demonstrated higher PA intensity, particularly during active periods, compared to the CI group, which exhibited significantly lower PA intensity (Supplementary Fig. 3a, 3b). The hourly average PA intensity revealed that the HC group maintained higher PA intensity during active periods (morning and afternoon) each day, while the CI group had relatively lower and less fluctuating PA levels (Supplementary Fig. 3b).

In the NHANES dataset, although the HC group still exhibited higher PA intensity than the CI group, the difference between the two groups was less pronounced compared to the NHATS dataset (Supplementary Fig. 3c, 3d). The hourly average PA intensity indicated that the HC group had higher PA intensity during active periods compared to the CI group, but the overall trends were more similar (Supplementary Fig. 3d). Across all time periods, the HC group consistently demonstrated higher PA intensity than the CI group, with this difference being more evident in the NHATS dataset.

### Recursive feature elimination and preliminary modeling analysis

RFE was performed on the 777 candidate features in the NHATS and NHANES datasets to identify the PA features most strongly associated with cognitive function. The top 20 features from each dataset were selected for subsequent machine learning modeling (Supplementary Tables 2 and Supplementary Table 3).

**Fig. 2:**
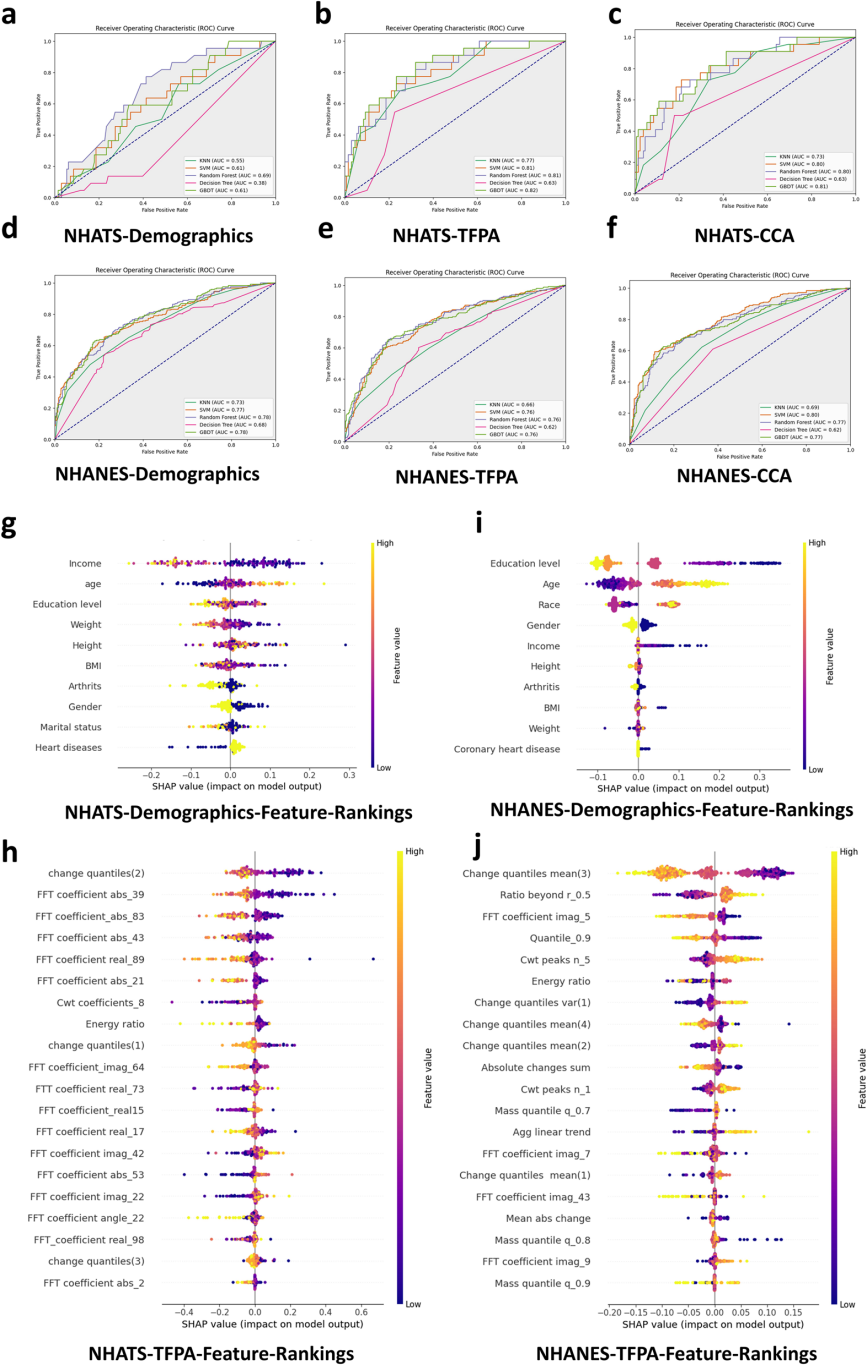
Preliminary Modeling Analysis and Interpretable Analysis of 20 PA Characteristic Modalities and Demographic and Disease History Modalities Based on RFE Screening

To assess the impact of independent PA features and demographic features on predictive performance, we constructed feature subsets using demographic features alone, PA features alone, and canonical correlation vectors fused from both. Five machine learning models were trained and evaluated on each subset. In the NHATS dataset, the highest AUCs were 0.69 for the demographic and disease history model (Fig. 2a), 0.82 for the PA model (Fig. 2b), and 0.81 for the fused model (Fig. 2c). Similarly, in the NHANES dataset, the highest AUCs were 0.79 for the demographic model (Fig. 2d), 0.76 for the PA-only model (Fig. 2e), and 0.80 for the fused model (Fig. 2f).

SHAP values were used to interpret the influence of demographic features on cognitive function prediction (Fig. 2g and i). In the NHATS dataset, income, age, and education level were the most important features, with income and education level positively correlated and age negatively correlated with cognitive function. In the NHANES dataset, education level, age, and race were the most important features, with education level positively correlated and age negatively correlated with cognitive function. The race feature suggested differences in cognitive function among racial groups. Income, education level, and age were the most important shared features across both datasets.

For PA features, the most important features in the NHATS dataset included Change quantiles, FFT coefficient abs, FFT coefficient real, Cwt coefficients, and FFT coefficient imag (Fig. 2h). In the NHANES dataset, the most important PA features included Change quantiles mean, Ratio beyond r, FFT coefficient imag, Quantile, and Cwt peaks n (Fig. 2j). Features from the Change quantiles, FFT coefficient, and CWT categories consistently ranked highly in both datasets, with Change quantiles ranking first in both independent PA features models.

### RCS and correlation analysis of PA Feature

**Fig. 3:**
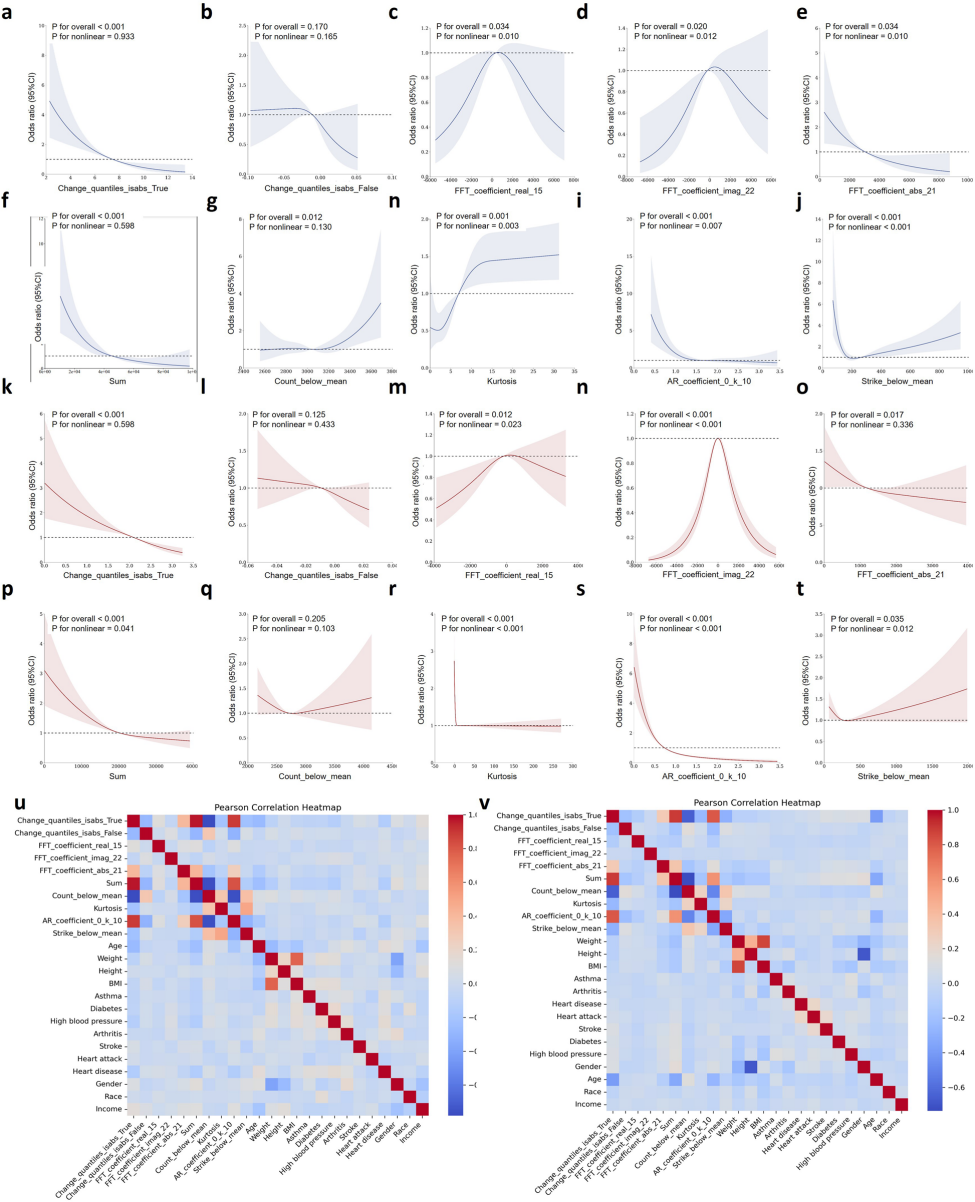
Feature correlation analysis based on RCS and Pearson correlation. (a-j) RCS curve analysis of the common 10 PA features in the NHATS dataset. (k-t) RCS curve analysis of the common 10 PA features in the NHANE dataset. (u) Heat map of Pearson correlations between demographic disease history variables and 10 PA characteristics in NHATS. (v) Heat map of Pearson correlations between demographic disease history variables and 10 PA characteristics in NHANES

After removing collinearity, the top 10 PA features jointly ranked by the post-hoc models (Supplementary Table 4) were selected for RCS analysis. Results indicate that higher change quantiles (when the parameter is True) are associated with a lower risk of CI in both datasets (NHATS: *p* < 0.001, NHANES: *p* < 0.001) (Fig. 3a and k), while no significant relationship exists when the parameter is False (Fig. 3b and i).

**Fig. 4:**
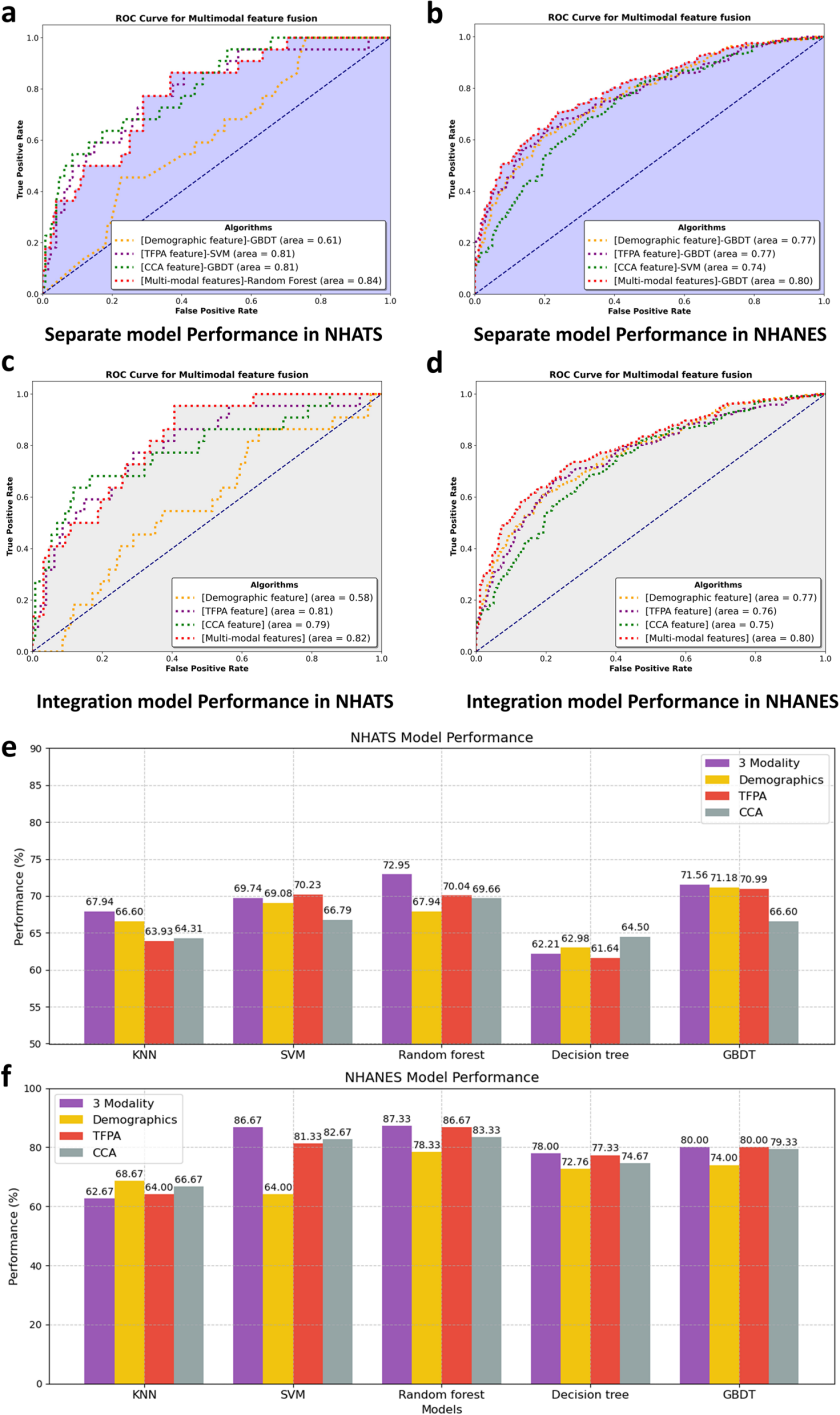
Model performance of CCA fusion-based secondary analysis of common PA features and demographics

FFT coefficients exhibit similar trends in NHATS (Fig. 3c and d) and NHANES (Fig. 3m and n) when the parameter is imag or real. CI risk is higher at intermediate FFT coefficient values and decreases at extreme values. FFT coefficients show significant nonlinear relationships in NHATS (FFT_imag: *p* = 0.02; FFT_real: *p* = 0.01) and similar trends in NHANES (FFT_imag: *p* < 0.001; FFT_real: *p* = 0.023). When the parameter is abs, higher FFT coefficients are associated with a lower CI risk (NHATS: *p* < 0.001, NHANES: *p* = 0.017) (Fig. 4e and o). Higher AR coefficients are associated with cognitive impairment (NHATS: *p* < 0.001, NHANES: *p* < 0.001) (Fig. 3i and s).

Regarding cumulative and statistical indicators, as the sum of PA intensity increases, CI risk decreases (NHATS: *p* < 0.001, NHANES: *p* < 0.001) (Fig. 3f and p). A significant nonlinear relationship exists between strike below mean values and CI risk, with increased risk and estimation uncertainty at higher values. This relationship is more significant in the NHATS dataset (*p* < 0.001), with dramatic changes at lower values and a significant increase in risk at higher values (Fig. 3j and t). The count below mean variable in NHATS has an overall significance of 0.012, but the nonlinear relationship is not significant (*p* = 0.130) (Fig. 3g). As the count below mean value increases, CI risk significantly increases, especially at higher values. This result is not significant in NHANES. Correlation analysis of the included features in both datasets (Fig. 3u and v) reveals that change quantiles and sum have strong positive correlations with AR coefficients and negative correlations with count below mean and age.

### Comprehensive Model Performance and subgroup analysis

Feature from demographics, PA, and CPV were integrated and modeled using five ML algorithms in both datasets. In the NHATS dataset, the random forest-based ML model achieved the highest AUC of 0.84 after combining data from the three modalities (Fig. 4a), while in the NHANES dataset, the GBDT-based ML model achieved the highest AUC of 0.80 (Fig. 4b).

To validate the predictive ability of the 10 selected common PA features, final modeling was performed using these features with the random forest model. The results showed an AUC of 0.82 in the NHATS dataset (Fig. 4c) and 0.80 in the NHANES dataset (Fig. 4d). The random forest model achieved the highest accuracy in both datasets, with the highest accuracy reaching 87.33% in NHATS (Fig. 4e and f).

A stratified analysis was conducted to assess the comprehensive model’s performance in mutually exclusive subgroups based on gender, age, and BMI. The model’s predictive performance was better in females than in males, particularly in NHATS (females: AUC = 0.92; males: AUC = 0.76) (Supplementary Fig. 4a, 4b). In the age-stratified analysis, the model performed better in the older adult group (age ≥ 80 years, AUC = 0.86) compared to the non-older adult group (AUC = 0.83) in the NHATS dataset. Conversely, in the NHANES dataset, the model’s performance was significantly better in the non-older adult group (AUC = 0.81) than in the older adult group (AUC = 0.67) (Supplementary Fig. 4c, 4d). In the BMI-stratified analysis, the model’s performance was better in the low BMI group (AUC = 0.84) than in the high BMI group (AUC = 0.68) in the NHATS dataset. However, in the NHANES dataset, the model’s performance was comparable between the high and low BMI groups (AUC = 0.75) (Supplementary Fig. 4e, 4f). To demonstrate the practical application of these findings, we developed a proof-of-concept system that implements these movement pattern analyses using wearable accelerometer data. Technical details and system architecture design of this implementation are provided in Supplementary File 1.

In subgroup analyses, we further explored the interactions between PA features and demographic characteristics. The interaction plots revealed distinct patterns between Change quantiles, FFT coefficients and demographic variables across datasets. In NHATS, higher Change quantiles values were associated with lower cognitive impairment probability across all demographic subgroups, with steeper slopes in females, older adults (≥ 80 years), and the high BMI group (≥ 28). Similarly, in NHANES, while maintaining the negative association trend, the interaction effects were more pronounced in males, younger adults (< 80 years), and the low BMI group (< 28) (Supplementary Figs. 5, 6).

## Discussion

This study demonstrated that PA patterns derived from wearable accelerometer data may serve as objective markers of cognitive status in older adults. By analyzing continuous activity data collected in natural settings, we identified several features that showed consistent associations with cognitive status across two independent cohorts. When combined with demographic information, these movement patterns provided meaningful insights into cognitive status, potentially offering a complementary approach to traditional cognitive assessments.

An advantage of PA pattern monitoring is its ability to provide continuous, objective data without learning effects or the need for repeated clinical visits. Traditional cognitive screening tools like MMSE, while well-validated, are limited by their point-in-time nature and susceptibility to practice effects when administered frequently. They also require trained personnel and standardized testing conditions, making regular monitoring challenging. In contrast, accelerometer-based PA pattern analysis can be conducted unobtrusively in natural settings over extended periods, potentially enabling earlier detection of subtle changes in daily functioning that may signal cognitive decline.

A key finding is that frequency-domain, time-domain, and nonlinear PA features play a crucial role in characterizing and predicting cognitive function, with higher importance than traditional PA indicators. Two features emerged as particularly significant: Change quantiles and FFT coefficients. Change quantiles measure the magnitude of activity intensity transitions over the 3-day period, with higher values indicating more frequent transitions between different activity intensities (e.g., from sitting to walking). FFT coefficients capture the rhythmicity of these activity changes, with higher values reflecting more regular activity patterns throughout the day.

Individuals with better cognitive function typically demonstrated more dynamic activity patterns, characterized by larger activity intensity transitions (higher Change quantiles) and more regular activity rhythms (stronger FFT coefficients). These patterns suggest better capacity for activity planning, execution, and adaptation - cognitive processes fundamental to executive function. In contrast, those with cognitive impairment often showed more monotonous activity patterns with fewer intensity variations and less regular rhythms.

Another major innovation of this study is the introduction of a CCA-based multimodal data modeling framework alongside mainstream machine learning algorithms. CCA reveals complex association patterns among multiple variable sets by mapping them into a shared latent space, enabling effective cross-modal data fusion and prediction [34]. The collaborative modeling of PA features and demographic factors achieved superior performance compared to other algorithms across three independent datasets, highlighting the value of cross-modal data integration in improving the accuracy and robustness of cognitive assessment.

This study explored and validated the potential of movement patterns as digital markers of cognitive status using large-scale, multi-center real-world data and advanced analytical methods. Our approach offers several notable strengths. First, we utilized two independent, nationally representative datasets, allowing for robust validation of our findings. Second, our analysis of continuous accelerometer data provided objective, real-world measurements of daily functioning, avoiding recall bias inherent in self-reported measures. Third, the consistency of key movement patterns across different cohorts suggests these markers may be generalizable across diverse populations.

Nevertheless, several limitations should be noted. First, while our findings suggest promising associations between movement patterns and cognitive status, these relationships were observed at specific time points and may not fully capture seasonal or longer-term variations in activity patterns. Second, the cognitive assessment methods differed between cohorts - while NHATS used a comprehensive assessment including physician diagnosis and validated screening tools, NHANES relied on cognitive performance tests with quartile-based cutoffs. Although this quartile-based approach has been used in previous studies, it may not accurately identify clinically significant cognitive impairment and could potentially misclassify individuals near the cutoff points. Third, neither dataset included biomarker data (e.g., cerebrospinal fluid markers, neuroimaging) that are increasingly recognized as important indicators for confirming cognitive impairment diagnosis. Furthermore, our sample may not be fully representative of the broader older adult population, particularly regarding racial and ethnic diversity, socioeconomic status, and geographical distribution. Fourth, while our use of minute-level features offered practical advantages for clinical applications - including alignment with natural activity cycles and reduced computational complexity for real-time monitoring - this approach has inherent limitations. Compared to raw accelerometer data (typically 30–100 Hz), minute-level aggregation may lose some fine-grained movement characteristics that could be relevant for specific motor function assessments. Additionally, the effectiveness of this approach heavily relies on appropriate feature engineering methods and understanding of temporal patterns. However, considering the practical requirements of cognitive assessment in older adults and the need for scalable monitoring solutions, we believe minute-level features strike a reasonable balance between feasibility and effectiveness, particularly for capturing function impairment movement patterns in daily life settings [35–37].

## Conclusions

This study identified consistent associations between specific movement patterns from wearable accelerometer data and cognitive status across two independent cohorts of older adults. Features capturing movement variability and rhythmicity, particularly change quintiles, provided meaningful information beyond traditional physical activity measures. When combined with demographic characteristics, these movement patterns demonstrated potential utility for cognitive monitoring in natural settings. The proof-of-concept application developed from these findings demonstrates the feasibility of translating movement pattern analysis into practical monitoring tools, offering a complementary approach to traditional cognitive assessments that provides continuous, objective data without the burden of repeated clinical testing.

## Supplementary Information

Supplementary Material 1: Supplementary figure 1. Participant selection flowchart

Supplementary Material 2: Supplementary figure 2. Detailed steps for machine learning model training and testing

Supplementary Material 3: Supplementary figure 3. Visualization of physical activity (PA) patterns in NHATS and NHANES datasets

Supplementary Material 4: Supplementary figure 4. Subgroup analysis results of the machine learning model

Supplementary Material 5: Supplementary figure 5. Interaction effects of key PA variables with gender, age, and BMI in the NHANES Dataset

Supplementary Material 6: Supplementary figure 6. Interaction effects of key PA variables with gender, age, and BMI in the NHATS Dataset

Supplementary Material 7: Supplementary table 1. Machine learning parameter settings based on GridSearchCV

Supplementary Material 8: Supplementary table 2. Feature contribution rankings in the NHATS dataset based on Recursive Feature Elimination

Supplementary Material 9: Supplementary table 3. Feature contribution rankings in the NHANES dataset based on Recursive Feature Elimination

Supplementary Material 10: Supplementary table 4. Commonly Selected Features in the NHATS and NHANES Datasets

Supplementary Material 11: Supplementary file 1. Modeling applications and systems development

## Data Availability

The original data for the study are available on the NHANES website: https:// wwwn.cdc.gov/nchs/nhanes/Default.aspx and NHATS website: https://www.nhats.org/researcher/nhats.

## Abbreviations

PA: Physical activity
MVPA: Moderate-vigorous physical activity
RFE: Recursive feature elimination
RCS: Restricted cubic splines
CCA: Canonical correlation analysis
CPV: Canonical projection vectors
FFT: Fast fourier transform
SHAP: Shapley additive explanations
