# Supplementary figures and images for: From physical activity patterns to cognitive status: development and validation of novel digital biomarkers for cognitive assessment in older adults
Source: Int J Behav Nutr Phys Act. 2025 Jan 20;22:11. doi: 10.1186/s12966-025-01706-x (PMC11748278; doi:10.1186/s12966-025-01706-x)

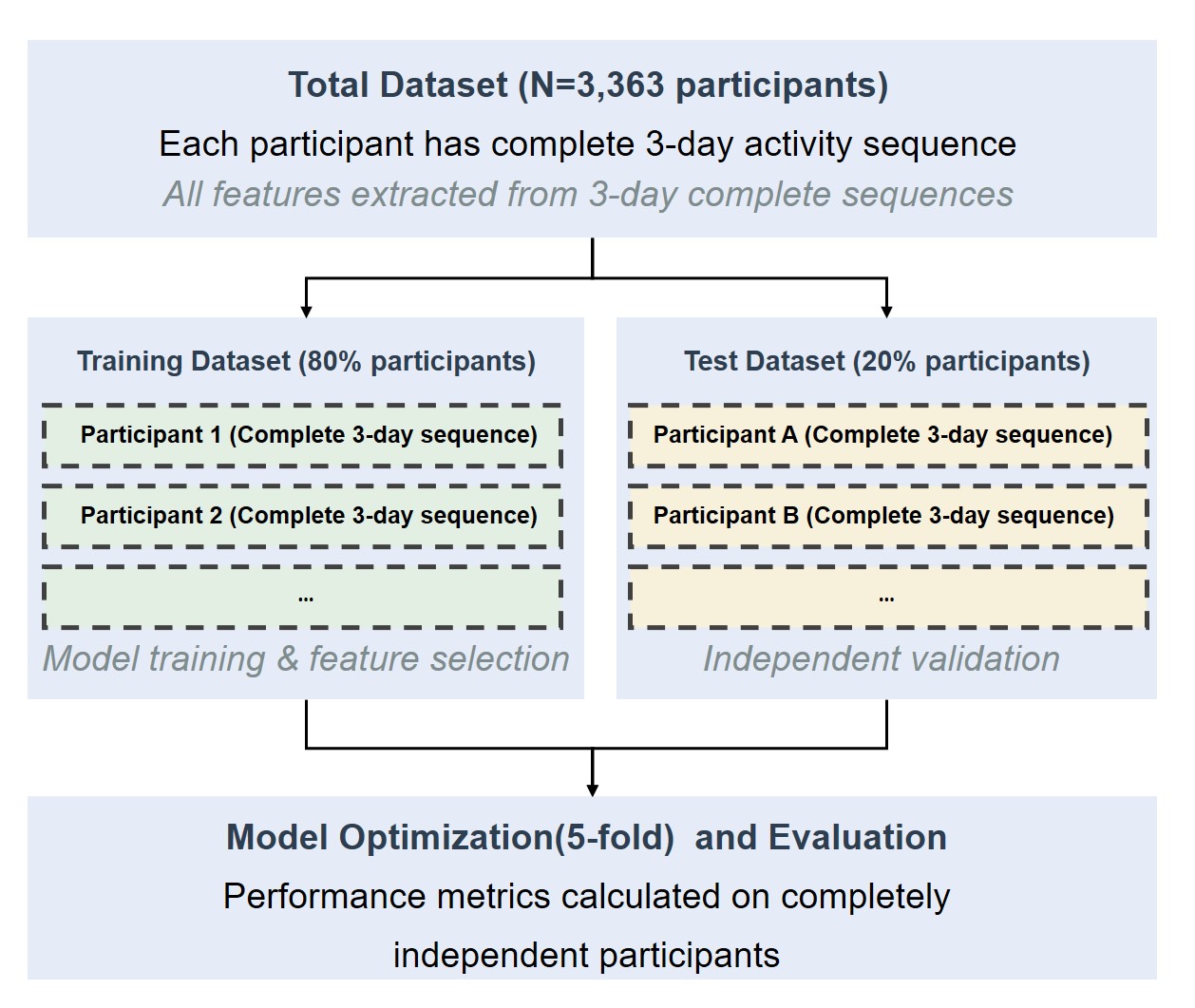

Supplement: Supplementary file 2 — Supplementary Material 2: Supplementary figure 2. Detailed steps for machine learning model training and testing [file 12966_2025_1706_MOESM2_ESM.jpg]

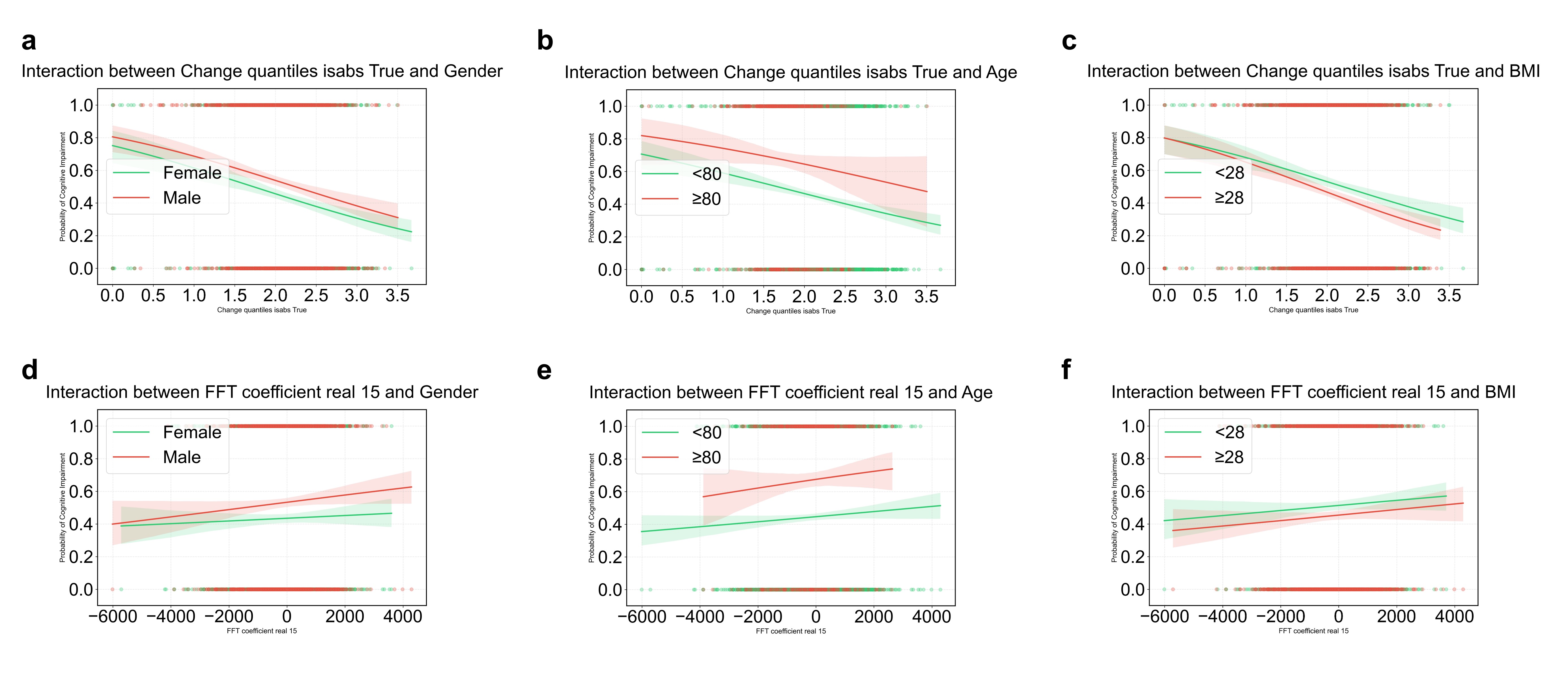

Supplement: Supplementary file 5 — Supplementary Material 5: Supplementary figure 5. Interaction effects of key PA variables with gender, age, and BMI in the NHANES Dataset [file 12966_2025_1706_MOESM5_ESM.jpg]

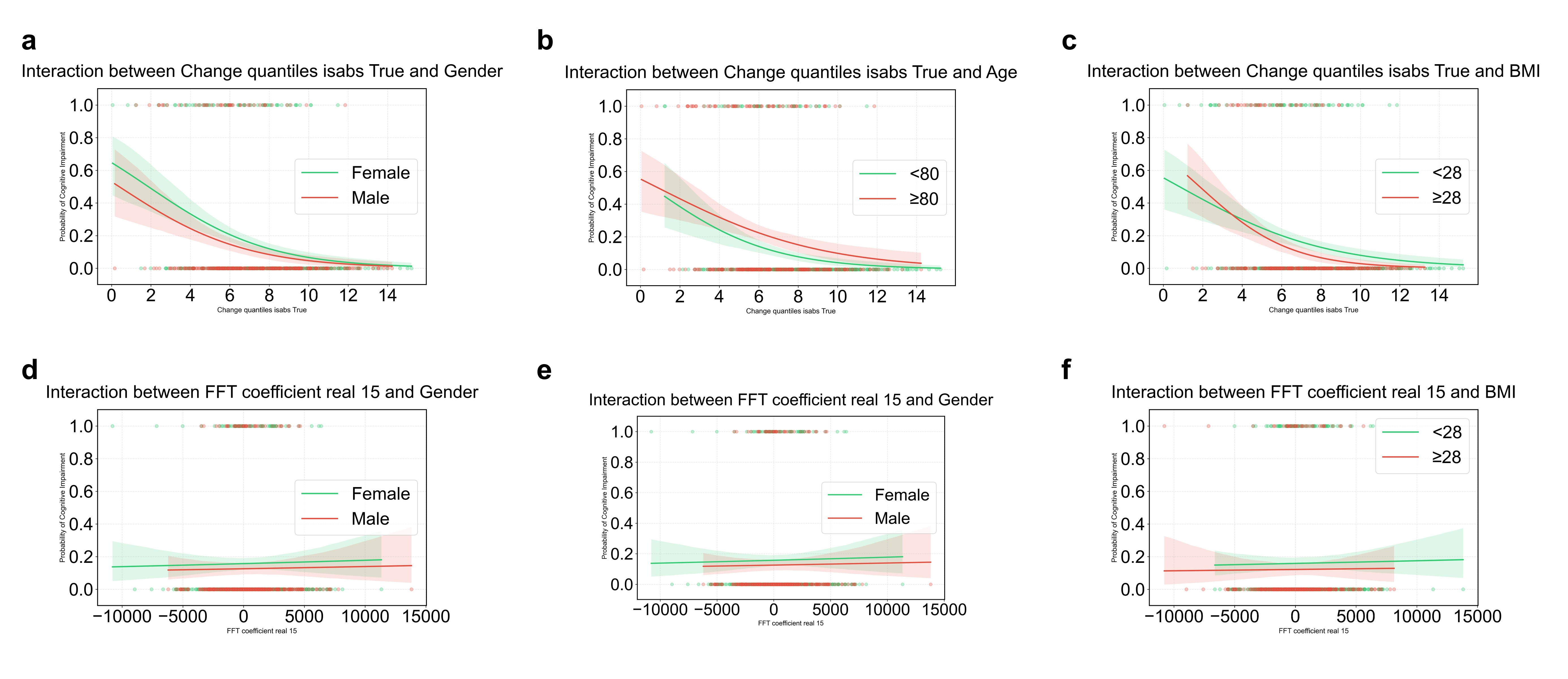

Supplement: Supplementary file 6 — Supplementary Material 6: Supplementary figure 6. Interaction effects of key PA variables with gender, age, and BMI in the NHATS Dataset [file 12966_2025_1706_MOESM6_ESM.jpg]
