# Supplementary material for: From physical activity patterns to cognitive status: development and validation of novel digital biomarkers for cognitive assessment in older adults
Source: Int J Behav Nutr Phys Act. 2025 Jan 20;22:11. doi: 10.1186/s12966-025-01706-x (PMC11748278; doi:10.1186/s12966-025-01706-x)
Supplement: Supplementary file 11 — Supplementary Material 11: Supplementary file 1. Modeling applications and systems development [file 12966_2025_1706_MOESM11_ESM.docx]

# Modeling Applications and Systems Development

An intelligent application system based on explainable artificial intelligence (XAI) was developed using a cloud-edge-end collaborative architecture for efficient data collection, analysis, and personalized service delivery. Mobile phone and smartwatch applications enable user interaction, device management, and data input at the edge (Figure 1D), allowing users to provide necessary information for personalized model prediction and analysis.

Lightweight data collection modules on edge devices collect PA data using built-in sensors, temporarily storing it locally before periodic upload to the cloud. This distributed approach enhances real-time performance and scalability. The cloud component, built on Tencent Cloud services, performs data processing and cognitive function evaluation using explainable machine learning models, generating personalized health insights and behavioral recommendations.

Firebase's cloud messaging service delivers personalized insights and recommendations to users' mobile devices, promoting engagement and compliance. User feedback and interaction continuously optimize the model's performance and user experience. The system's architecture allows for seamless integration of data from various sources, providing a comprehensive view of an individual's health status and cognitive function.

The developed system leverages explainable machine learning models to offer novel means for assessing and intervening in the cognitive function of older adults, with potential applications in smart elderly care and precision medicine. The explainable nature of the models ensures transparency and trust in the system's predictions and recommendations. The cloud-edge-end collaborative architecture enables scalable deployment and efficient resource utilization, making the system suitable for large-scale implementation and future advancements in technology and research.
